# Synaptic connectivity of the cholinergic axons in the olfactory bulb of the cynomolgus monkey

**DOI:** 10.3389/fnana.2015.00028

**Published:** 2015-03-17

**Authors:** Teresa Liberia, José Miguel Blasco-Ibáñez, Juan Nácher, Emilio Varea, José Luis Lanciego, Carlos Crespo

**Affiliations:** ^1^Faculty of Biology, Department of Cell Biology, University of ValenciaBurjassot, Valencia, Spain; ^2^Neurosciences Division, Center for Applied Medical Research (CIMA), University of NavarraPamplona, Spain; ^3^Centro de Investigación Biomédica en Red sobre Enfermedades Neurodegenerativas (CIBERNED)Pamplona, Navarra, Spain; ^4^Instituto de Investigaciones Sanitarias de Navarra (IdiSNA)Pamplona, Navarra, Spain

**Keywords:** olfaction, acetylcholine, synapses, non-human primates, interneurons

## Abstract

The olfactory bulb (OB) of mammals receives cholinergic afferents from the horizontal limb of the diagonal band of Broca (HDB). At present, the synaptic connectivity of the cholinergic axons on the circuits of the OB has only been investigated in the rat. In this report, we analyze the synaptic connectivity of the cholinergic axons in the OB of the cynomolgus monkey (*Macaca fascicularis*). Our aim is to investigate whether the cholinergic innervation of the bulbar circuits is phylogenetically conserved between macrosmatic and microsmatic mammals. Our results demonstrate that the cholinergic axons form synaptic contacts on interneurons. In the glomerular layer, their main targets are the periglomerular cells, which receive axo-somatic and axo-dendritic synapses. In the inframitral region, their main targets are the granule cells, which receive synaptic contacts on their dendritic shafts and spines. Although the cholinergic boutons were frequently found in close vicinity of the dendrites of principal cells, we have not found synaptic contacts on them. From a comparative perspective, our data indicate that the synaptic connectivity of the cholinergic circuits is highly preserved in the OB of macrosmatic and microsmatic mammals.

## Introduction

It is widely known that the olfactory bulb (OB) of mammals receives projections from cholinergic neurons located in the horizontal limb of the diagonal band of Broca (HDB; Broadwell and Jacobowitz, 1976; Godfrey et al., 1980; Macrides et al., 1981; Mesulam et al., 1983; Carson, 1984; Rye et al., 1984; Záborszky et al., 1986). Abundant literature demonstrates that the distribution of the cholinergic axons throughout the OB shows a laminar pattern, which remains constant in all the mammalian species analyzed so far, including macrosmatic animals such as rats (Godfrey et al., 1980; Zheng et al., 1987; Nickell and Shipley, 1988; Ojima et al., 1988; Le Jeune and Jourdan, 1991; Butcher et al., 1992; Phelps et al., 1992; Kasa et al., 1995; Arvidsson et al., 1997; Ichikawa et al., 1997; Gómez et al., 2005), mice (Carson and Burd, 1980; Weruaga et al., 2001; Salcedo et al., 2011), hamsters (Macrides et al., 1981), cats (Kimura et al., 1981), rabbits (Chao et al., 1982) or hedgehogs (Crespo et al., 1999) and microsmatic species such as monkeys (Porteros et al., 2007) or humans (Kovacs et al., 1998).

The cholinergic axons innervate all the layers of the OB, with the exception of the olfactory nerve layer. The glomerular layer and the internal plexiform layer are the strata that contain the highest density of cholinergic fibers (Carson and Burd, 1980; Godfrey et al., 1980; Macrides et al., 1981; Crespo et al., 1999; Porteros et al., 2007). This distribution pattern suggests that the cholinergic modulation of the bulbar circuits occurs at two different levels. On the one hand, the cholinergic axons may influence the entrance of sensory information at the level of the olfactory glomeruli (Nickell and Shipley, 1988; Crespo et al., 2000). On the other hand, they may modulate the status of inhibition/disinhibition of the principal cells, influencing the activity of the granule cells at the level of the internal plexiform and granule cell layers (Nickell and Shipley, 1988; Crespo et al., 2000).

It is widely accepted that cholinergic neurons exert their actions in the central nervous system through synaptic and extrasynaptic mechanisms. At present, the synaptic connectivity of the cholinergic axons on the circuits of the OB has only been investigated in the rat (Le Jeune and Jourdan, 1993; Kasa et al., 1995). By combining choline acetyltransferase (ChAT) immunohistochemistry and electron microscopy, Kasa et al. (1995) demonstrated that the cholinergic axons establish asymmetrical synaptic contacts on the dendrites of some bulbar interneurons, these including periglomerular cells, granule cells and superficial short-axon cells. On the contrary, they did not find synaptic contacts from cholinergic fibers on principal cells.

Unfortunately, there are no data describing the synaptic connectivity of cholinergic fibers on the bulbar circuits of microsmatic species. In this context, our aim here is to investigate the synaptic connectivity of the cholinergic axons on the circuits of the OB of the cynomolgus monkey (*Macaca fascicularis*). For this purpose, we have combined immunohistochemical techniques with electron microscopy. Previous reports have demonstrated that the vesicular acetylcholine transporter (VAChT) appears in the preterminal- and terminal-like portions of cholinergic axons (Arvidsson et al., 1997; Ichikawa et al., 1997). Therefore, we have used antibodies against VAChT in order to stain the presynaptic elements of the cholinergic synapses.

## Material and Methods

### Animals and Tissue Processing

The OBs from four adult male cynomolgus monkeys (*Macaca fascicularis*) were used in this study. Animal handling was conducted in accordance with the guidelines of the European Parliament and the Council of the European Union (Directive 2010/63/EU) and conformed to the Spanish legislation for the use and care of animals (RD1201/2005). The experimental design was approved by the Ethical Committee for Animal Testing of the University of Navarra (ref: 018/2008).

The animals were anesthetized with an overdose of chloral hydrate and perfused transcardially with a saline Ringer solution followed by 3,000 ml of a fixative solution containing 4% paraformaldehyde and 0.3% glutaraldehyde in 0.125 M phosphate buffer, pH 7.4 (PB). Perfusion was continued with 1,000 ml of a cryoprotectant solution containing 10% glycerin and 2% dimethylsulfoxide in PB. After perfusion, the skull was opened, the brain removed and the OBs dissected out and stored at −80°C in a cryoprotectant solution containing 20% of glycerin and 2% dimethylsulfoxide in PB. For the execution of the experiments, the temperature of the OBs was raised from −80°C to +20°C and then, the cryoprotectant solution was removed and replaced with PB. After being thoroughly rinsed in PB, 60-μm-thick coronal sections through the OBs were obtained on a vibratome and collected in PB.

Since the animals were fixed with a fixative that contained glutaraldehyde, sections were pre-treated with 1% sodium borohydride in PB for 20 min before the immunohistochemical staining. The sections used for electron microscopy processing were cryoprotected by immersion in a mixture of 25% sucrose and 10% gycerol in 0.1 M PB and freeze–thawed three times in liquid nitrogen in order to enhance antibody penetration. Finally, all sections were processed for immunocytochemistry.

### Pre-Embedding Immunocytochemical Detection of VAChT for Light and Electron Microscopy

The commercial sources for all the antibodies used in this study are summarized in Table 1.

**Table 1:** **Antibodies used in the study**.

Antibody	Host	Company	Reference	Dilution
Anti-VAChT	Goat	Phoenix Pharmaceuticals	#H-V007	1:10,000
Anti-CR	Rabbit	Swant	#7699/3H	1:5,000
Anti-TH	Rabbit	Millipore	#AB 152	1:1,000
Anti-goat	Horse	Vector Labs	#BA-9500	1:200
Anti Rabbit	Goat	Thermo Scientific	#31820	1:200

The detection of VAChT for light and electron microscopy was performed using the avidin-biotin-peroxidase (ABC) method. According to this procedure, sections were sequentially incubated as follows: (a) Blocking solution containing 10% normal horse serum (NHS) and 0.05% sodium azide in PB, for 60 min at room temperature. 0.1% Triton X-100 was added to the blocking solution when the sections were destined to light microscopy; (b) Goat anti-VAChT antibody (Phoenix Pharmaceuticals #H-V007) diluted 1:10,000 in PB containing 1% NHS and 0.05% sodium azide, for 48 h at 4°C. 0.1% Triton X-100 was added when the sections were destined to light microscopy; (c) Biotinylated horse anti-goat IgG (Vector Labs. Burlingame, CA; USA) diluted 1:200 in PB, for 2 h at room temperature; (d) Avidin-biotinylated horseradish peroxidase complex (ABC; Vector Labs.) diluted 1:200 in PB, for 2 h at room temperature. After each step, sections were carefully rinsed in PB (3 × 10 min). Finally, the peroxidase reaction was developed using 0.05% 3,3-diaminobenzidine tetrahydrochloride (DAB; Sigma-Aldrich, St. Louis, MO, USA) as chromogen and 0.003% hydrogen peroxide in PB. The reaction was developed at room temperature until the specific VAChT-immunostaining could be visualized under the light microscope. Then, sections were carefully rinsed in PB (3 × 10 min), treated with 1% osmium tetroxide (Electron Microscopy Sciences, Hatfield, PA, USA) containing 7% glucose in PB for 45 min at room temperature and washed repeatedly in PB. Then, sections destined to electron microscopy were stained with 1% uranyl acetate (Electron Microscopy Sciences) in maleate buffer, pH 4.5, for 90 min at 4°C. Finally, all sections were dehydrated through graded ethanol series, cleared in propylene oxide and flat-embedded in Durcupan (ACM, Fluka AG, Switzerland) between slides and coverslips. Durcupan was polymerized overnight at 60°C. Flat-embedded sections were examined under light microscopy. After exhaustive analysis, some fields from the layers containing VAChT-positive profiles were re-embedded in Durcupan for further analyses under electron microscopy. The re-embedded sections were cut on an ultramicrotome, and serial 60-nm-thick ultrathin sections were obtained and mounted on single-slot Formvar-coated nickel grids. The ultrathin sections were stained with lead citrate and analyzed under the electron microscope.

Controls were carried out by omitting either the primary or the bridge antibodies in each step or by incubating some sections exclusively in 0.05% DAB and 0.003% hydrogen peroxide in PB, in order to rule out the presence of endogenous peroxidase activity in the tissue and to assess the specificity of the immunohistochemical method. Non-specific stain was never found in these controls.

### Double Immunocytochemistry for VAChT and TH/CR for Light Microscopy

In order to identify the neurochemical identities for the targets of the VAChT-containing fibers in the glomerular layer, we carried out a sequential double immunocytochemical staining for VAChT combined with two neuroanatomical markers that stain specifically two populations of periglomerular cells. Selected markers were the enzyme tyrosine hydroxylase (TH) and the calcium binding protein calretinin (CR). Sections were first processed for the detection of VAChT as follows: (a) Blocking solution containing 10% NHS, 0.1% Triton X-100 and 0.05% sodium azide in PB, for 60 min at room temperature; (b) Goat anti- VAChT antibody diluted 1:10,000 in PB containing 1% NHS, 0.1% Triton X-100 and 0.05% sodium azide, for 48 h at 4°C; (c) Biotinylated horse anti-goat IgG diluted 1:200 in PB, for 2 h at room temperature; (d) ABC complex diluted 1:200 in PB, for 2 h at room temperature. After each step, sections were carefully rinsed in PB (3 × 10 min). Then, the peroxidase reaction was developed using 0.05% DAB intensified by 0.5 M ammonium-nickel sulphate (DAB-Ni) as chromogen and 0.003% hydrogen peroxide in PB. The DAB-Ni chromogen provides a black reaction product. The reaction was developed until the specific VAChT-immunostaining was clearly visible under light microscopy. After carefully rinsing the VAChT-immunolabelled sections in cold PB (4°C), they were processed for the second immunocytochemistry in order to detect CR or TH. Before the second immunocytochemistry, sections were treated with 0.05% sodium azide in PB in order to block putative residual peroxidase activity from the first immunocytochemistry. For this second immunocytochemistry, sections were sequentially incubated in: (a) polyclonal rabbit anti-CR IgG (Swant, 1:5,000) or rabbit anti-TH IgG (Millipore, 1:1,000) in PB containing 0.1% Triton X-100 and 0.05% sodium azide, for 48 h at 4°C; (b) Biotinylated goat ant-rabbit IgG (Thermo Scientific) diluted 1:200 in PB, for 2 h at room temperature. (c) ABC complex diluted 1:200 in PB, for 2 h at room temperature. After each step, sections were rinsed in PB. Then, the peroxidase reaction was developed using 0.05% DAB as chromogen and 0.003% hydrogen peroxide in PB, until specific immunostaining was clearly observed, providing a brown precipitate. Finally, sections were washed in PB (3 × 10 min), mounted on gelatin-coated slides, air drayed, dehydrated through graded ethanol series, cleared in xylene and coverslipped with Eukitt (Kindler GmbH, Freiburg, Germany).

Omission of the primary or bridge antibodies in each step and incubation of sections exclusively in 0.05% DAB-Ni or DAB and 0.003% hydrogen peroxide were used as controls. No residual activity was found in these controls.

## Results

### Distribution of VAChT-Containing Elements

The distribution of the VAChT-containing elements in the macaque OB shows a laminar pattern similar to that previously described using other cholinergic markers, such as ChAT and acetylcholinesterase (Porteros et al., 1997). The VAChT-containing elements were always identified as varicose axons and puncta. Neither neuronal cell bodies nor dendritic trunks showed VAChT-immunoreactivity.

**Figure 1:**
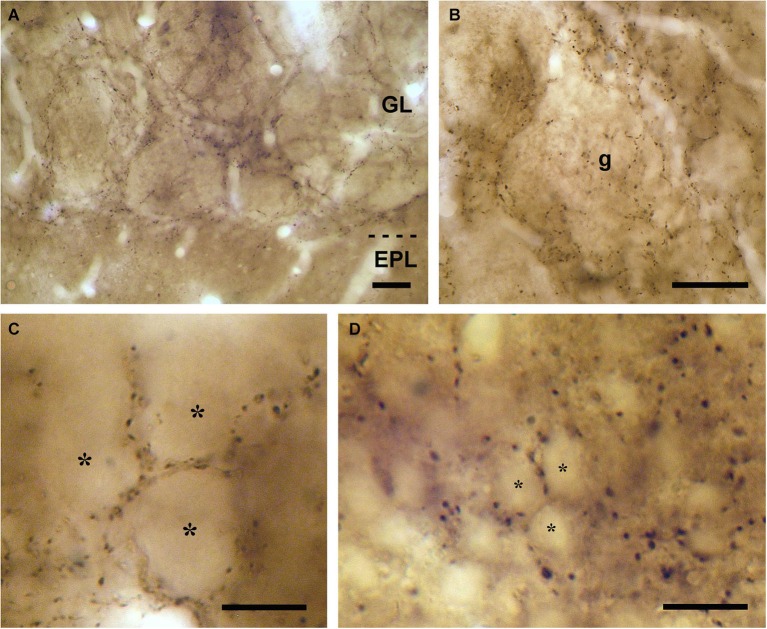
**VAChT-staining in the glomerular layer under light microscopy. (A)** Low-magnification view of the glomerular layer (GL) showing VAChT-containing puncta. Note the low density of staining found in the external plexiform layer (EPL). **(B)** Detailed view of an olfactory glomerulus (g). The VAChT-containing fibers run throughout the periglomerular region and also innervate the glomerular neuropil. **(C)** High-magnification view of the neuropil of an olfactory glomerulus revealing that the VAChT-containing puncta appear restricted to strands surrounding neuropil compartments where no VAChT-containing puncta are found (asterisks). **(D)** High-magnification photomicrograph taken from the periglomerular region of the glomerular layer showing the somata of immunonegative neurons (asterisks) surrounded by VAChT-containing puncta. Scale bars: 30 μm in **(A)** and **(B)**; 15 μm in **(C)** and **(D)**.

All the bulbar layers contained VAChT-positive axons, with the exception of the olfactory nerve layer. The highest density of VAChT-positive fibers was found in the glomerular layer (Figure 1). Here, the cholinergic fibers spread throughout the periglomerular region surrounding the olfactory glomeruli. Axon collaterals arose from these fibers and innervated the glomerular neuropil (Figures 1A,B). The distribution of the cholinergic axons inside the glomeruli was not homogeneous: They were restricted to some strands of neuropil and were never found in other neuropil compartments (Figure 1C). The neuropil of the olfactory glomeruli is divided into two separate compartments as previously described (Kosaka et al., 1997; Liberia et al., 2013): The “olfactory nerve zone”, which contains the axons of the olfactory nerve, and the “non-olfactory nerve zone”, which does not contain axons of the olfactory nerve. The analysis of the glomerular neuropil under electron microscopy demonstrated that the distribution of the VAChT-containing axons was restricted to the “non-olfactory nerve zone” and they did not innervate the “olfactory nerve zone” (data not shown).

**Figure 2:**
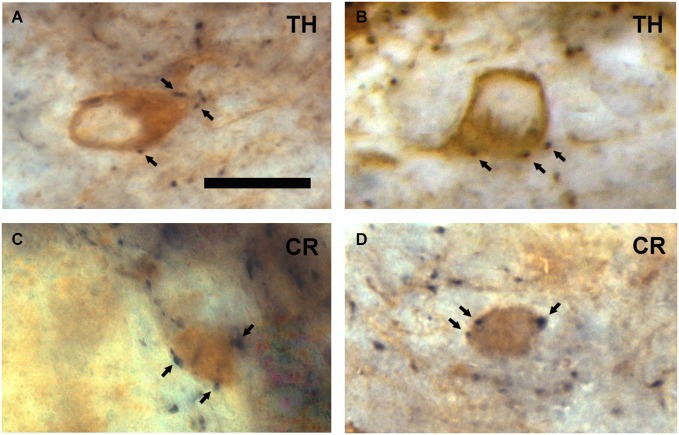
**Neurochemical characterization of the neurons surrounded by VAChT-containing puncta in the periglomerular region of the glomerular layer. (A,B)** Examples showing that TH-containing periglomerular cells (brown DAB precipitate) are surrounded by VAChT-containing puncta (black DAB-Ni precipitate; arrows). **(C,D)** Panels illustrating that CR-containing periglomerular cells (brown DAB precipitate) are surrounded by VAChT-containing puncta (black DAB-Ni precipitate; arrows). Scale bar: 10 μm.

In the periglomerular region, the VAChT-containing puncta were frequently found surrounding the cell bodies of some small juxtaglomerular neurons (Figure 1D). The size of these neurons (they ranged from 7 to 10 μm; *n* = 100) resembled that of the periglomerular cells previously described in the macaque OB (Liberia et al., 2013). In order to investigate whether they were, indeed, periglomerular cells, we combined the detection of VAChT with the detection of two neurochemical markers that stain specifically two subsets of periglomerular cells: The enzyme TH and the calcium binding protein CR. TH stains a population of type 1 periglomerular cells and CR stains a population of type 2 periglomerular cells in the macaque OB as previously described (Liberia et al., 2013). The DAB/DAB-Ni double immunocytochemical method was used for these experiments. We found VAChT-containing puncta surrounding both TH- and CR-containing periglomerular cells (Figure 2).

**Figure 3:**
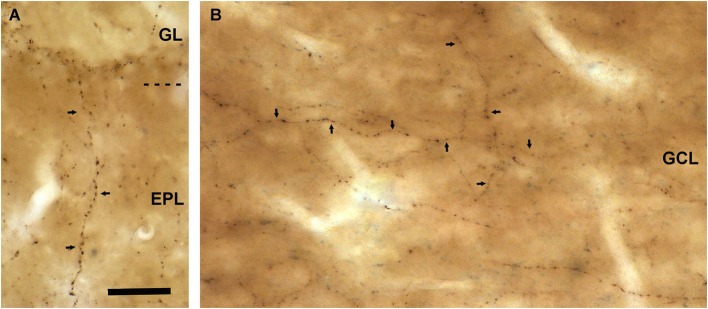
**VAChT-staining in the external plexiform layer and in the inframitral region under light microcopy. (A)** A varicose axon containing VAChT (arrows) crosses the external plexiform layer (EPL) perpendicularly to the bulbar lamination. The glomerular layer (GL) is shown in the upper side of the micrograph. **(B)** VAChT-containing fibers (arrows) running throughout the superficial portion of the granule cell layer (GCL). Note that some fibers run parallel and others perpendicularly to the bulbar lamination. Scale bar: 30 μm.

The external plexiform layer contained some VAChT-positive axons. Many of them were found ascending from the inframitral region. They crossed the external plexiform layer perpendicularly to the lamination of the OB and reached the glomerular layer (Figure 3A). The inframitral region, which includes the internal plexiform layer and the granule cell layer, contained a high density of VAChT-positive axons. Most of them were oriented in parallel to the lamination of the OB and distributed within the rows of granule cells (Figure 3B). However, some axons were oriented perpendicularly to the bulbar lamination, crossing the mitral cell layer and ascending towards the external plexiform layer.

### Connectivity of the VAChT-Containing Boutons

**Figure 4:**
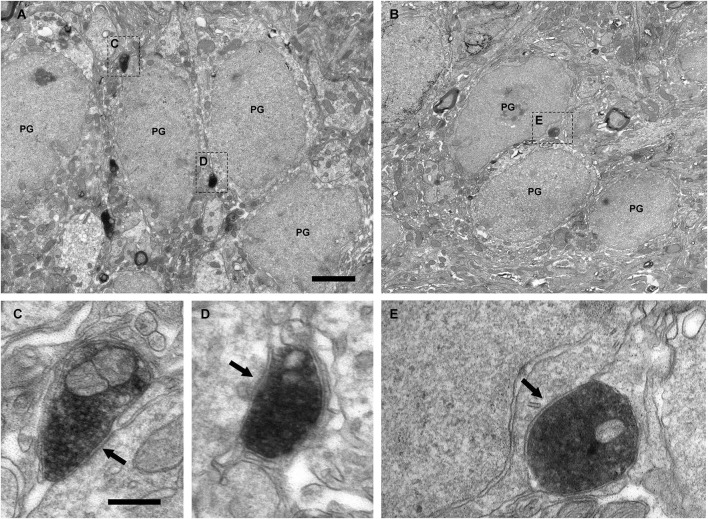
**Perisomatic innervation of periglomerular cells by VAChT-containing boutons under electron microscopy. (A,B)** Low-magnification views of the periglomerular region of the glomerular layer showing the somata of periglomerular cells (PG) surrounded by VAChT-containing boutons. The squared boutons are shown at higher magnification in the panels **(C–E). (C–E)** Synaptic contacts (arrows) from the VAChT-containing boutons on the somata of periglomerular cells. Scale bars: 2 μm in **(A)** and **(B)**; 200 nm in **(C), (D)** and **(E)**.

Electron microscopy examination demonstrated that the VAChT-containing axons formed axo-dendritic and axo-somatic synaptic contacts on some bulbar neurons. The vast majority of the cholinergic synapses did not show evident electron-dense postsynaptic thickenings. Therefore, they were classified morphologically as symmetrical synapses (Figures 4, 6–8, 9A,B). However, few synaptic contacts showed an ambiguous postsynaptic thickening and their symmetric or asymmetric nature was doubtful (Figures 9C,D). The DAB-precipitate filled up the VAChT-containing axons and thus hampered a detailed analysis of their ultrastructural features. However, presynaptic boutons containing numerous medium-sized to large round synaptic vesicles and some mitochondria were clearly observed (Figures 4, 6–9).

**Figure 5:**
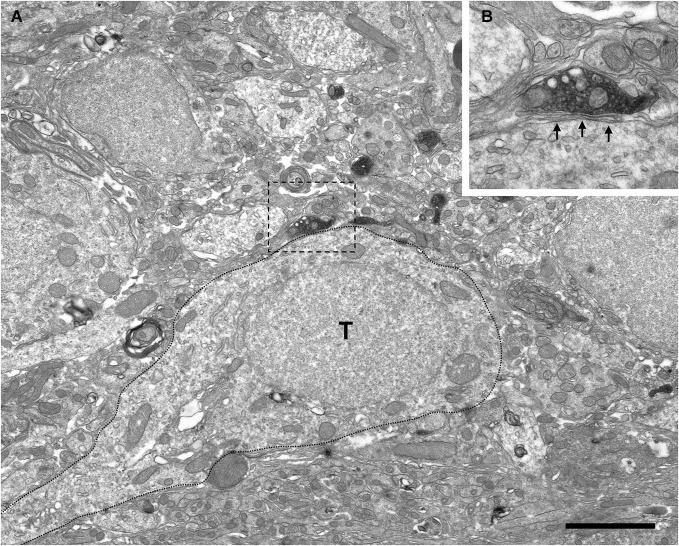
**Absence of perisomatic synaptic contacts from VAChT-containing boutons on external tufted cells. (A)** Low-power magnification view of the periglomerular region of the glomerular layer under electron microscopy showing VAChT-containing boutons close to the soma of an external tufted cell (T; dotted line). The square bouton is magnified in the panel **(B). (B)** The VAChT-containing bouton does not form synaptic contacts on the soma of the external tufted cell. Glial lamellae separate the plasma membranes of the VAChT-containing axon and the external tufted cell (arrows). Scale bar: 2 μm.

**Figure 6:**
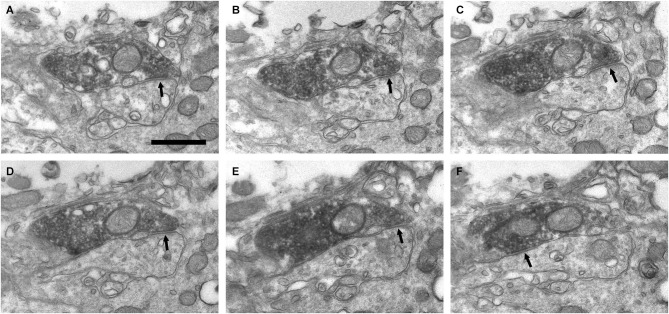
**Connectivity of the VAChT-containing boutons in the periglomerular region of the glomerular layer. (A–F)** Series of ultrathin sections showing symmetrical synaptic contacts (arrows) from a VAChT-containing axon on a dendritic spine. Scale bar: 500 nm.

**Figure 7:**
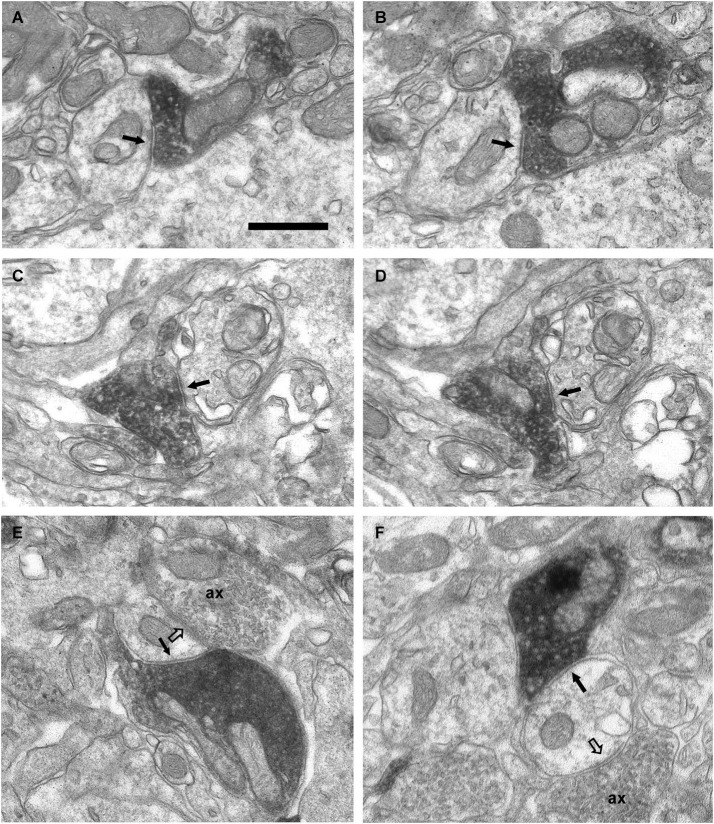
**Axo-dendritic synaptic contacts from VAChT-containing boutons in the glomerular layer. (A,B)** Serial sections showing a symmetrical synaptic contact (arrows) from a VAChT-containing axon on the dendritic shaft of a neuron located in the periglomerular region of the glomerular layer. **(C,D)** Serial sections showing the symmetrical synaptic contact (arrows) from a VAChT-containing axon on the dendritic appendage of a neuron located in the neuropil of an olfactory glomerulus. **(E,F)** Axo-dendritic symmetrical synaptic contacts from VAChT-containing boutons (arrows). The postsynaptic dendrites receive additional asymmetrical synaptic contacts (open arrows) from non-cholinergic axons (ax). Scale bar: 500 nm.

**Figure 8:**
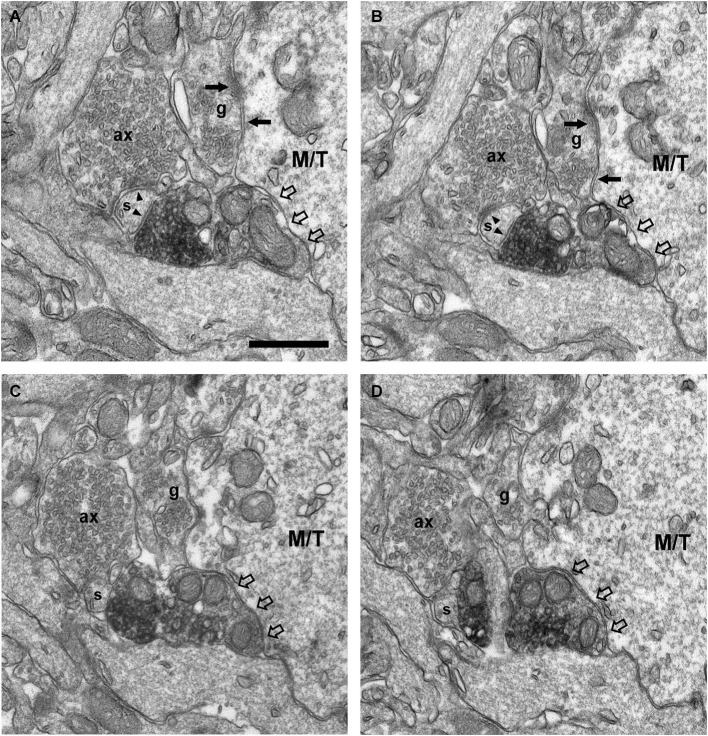
**Connectivity of the VAChT-containing boutons in the external plexiform layer. (A–D)** Series of ultrathin sections showing the connectivity of a VAChT-containing bouton in the external plexiform layer. A dendritic spine (s) receives two symmetrical synaptic contacts (arrowheads): One from a VAChT-containing bouton (electrondense DAB precipitate) and other from a non-cholinergic axon (ax). Note that, although the VAChT-containing bouton is beside the large dendrite of a principal cell (M/T), there are no synaptic contacts from the cholinergic axon on the principal cell (open arrows). Arrows point to dendro-dendritic reciprocal synaptic contacts between the principal cell and the gemmule of a granule cell (g). Scale bar: 500 nm.

**Figure 9:**
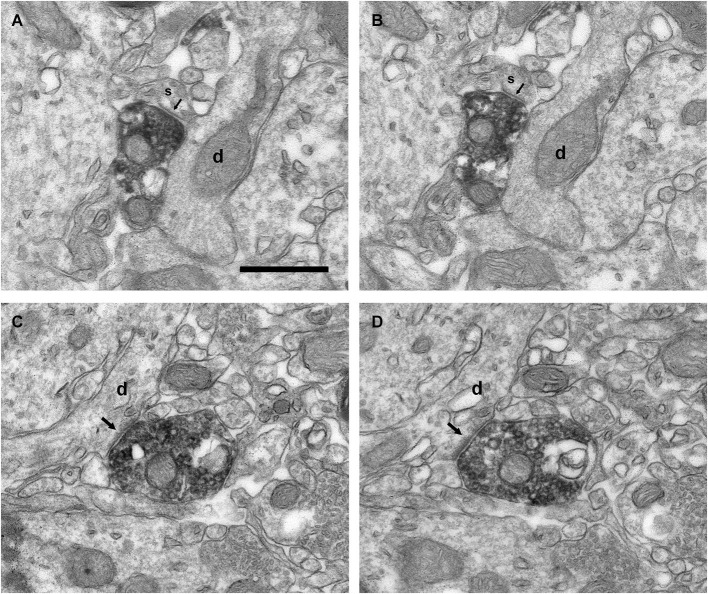
**Connectivity of the VAChT-containing boutons in the inframitral region. (A,B)** Serial sections showing the symmetrical synaptic contact (arrows) from a VAChT-containing bouton on the spine (s) of a dendrite (d) of a granule cell located in the granule cell layer. **(C,D)** Serial sections showing the asymmetrical synaptic contact (arrows) from a VAChT-containing bouton on the dendritic shaft (d) of a granule cell located in the granule cell layer. Scale bar: 500 nm.

The electron microscopic analysis of the glomerular layer demonstrated that the neurons of the periglomerular region innervated by cholinergic boutons had all the ultrastructural features that typically characterize periglomerular cells: Their nuclei almost filled the entire cell bodies, leaving just a thin ring of cytoplasm surrounding the nuclear region, and they contained deep nuclear indentations. The cholinergic boutons formed synaptic contacts on the somata of these interneurons (Figure 4). On the contrary, we never found synaptic contacts from the cholinergic axons on the somata of tufted cells. Indeed, when a cholinergic bouton was found close to the cell body of a tufted cell, thin lamellae of glia were observed between both elements, preventing the establishment of synaptic contacts (Figure 5).

In addition to the perisomatic innervation of some periglomerular cells, we have found synapses from cholinergic axons on dendrites located in the periglomerular region of the glomerular layer as well as in the neuropil of the olfactory glomeruli. Some synaptic contacts were found on the dendritic shafts and some others on dendritic appendages similar to spines or gemmules (Figures 6, 7). The dendritic profiles that received synapses from the VAChT-containing boutons were also engaged in other non-cholinergic synaptic contacts: They were frequently found receiving asymmetrical synaptic contacts from axons of unidentified origin (Figures 7E,F).

Since a few VAChT-containing axons ascended from the inframitral region and crossed the external plexiform layer (Figure 3A), we have analyzed their connectivity in this layer (Figure 8). In the external plexiform layer, the cholinergic axons ran frequently along the dendrites of mitral and tufted cells. However, we have found no synaptic contacts from the cholinergic boutons on the dendrites of mitral and tufted cells. The unique synaptic contacts from VAChT-containing boutons that we have found in this layer were located on small dendritic appendages similar to spines. Sometimes, the appendages that received the cholinergic synapses also received non-cholinergic synapses from axons of unidentified origin (Figure 8). The morphology of the spines and their connectivity resembled the morphology and connectivity of the spines of granule cells previously reported (Price and Powell, 1970b).

The ultrastructural features of the VAChT-containing boutons located in the internal plexiform layer and in the granule cell layer were identical to those described above for the boutons located in the glomerular layer. All of these boutons made synaptic contacts on thin dendrites, which were frequently oriented perpendicularly to the lamination of the OB (Figure 9). The contacts were found on appendages similar to spines (Figures 9A,B) or on the dendritic shafts (Figures 9C,D). The morphology of the dendrites, their orientation, and their synaptic connectivity were identical to the morphology, orientation and connectivity of the dendrites of granule cells described by Price and Powell (1970a,b).

## Discussion

**Figure 10:**
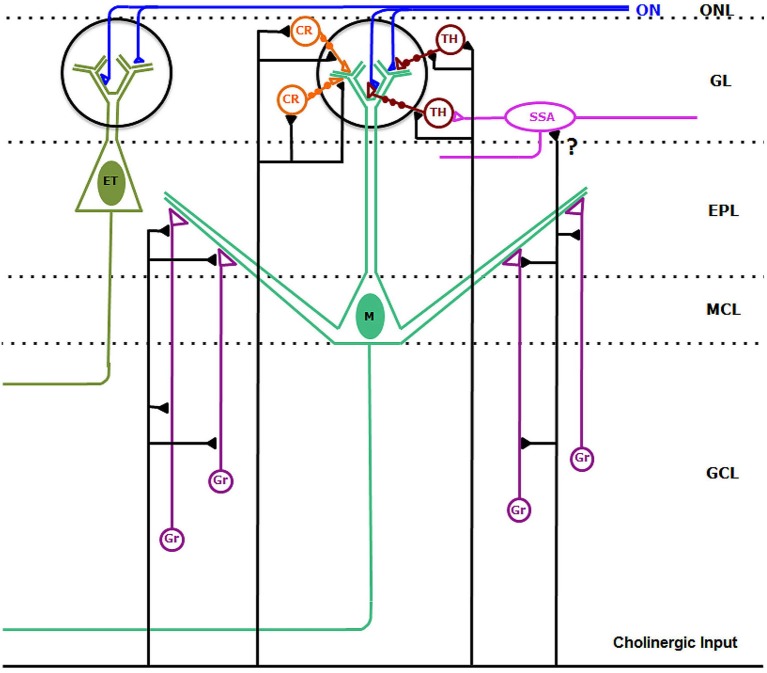
**Synaptic connectivity of the cholinergic circuits in the macaque olfactory bulb**. Cholinergic axons establish synaptic contacts on bulbar interneurons. On the one hand, cholinergic fibers form synaptic contacts on the somata, dendrites and dendritic appendages of type 1 (TH-containing) and type 2 (CR-containing) periglomerular cells. In addition, cholinergic axons form synaptic contacts on the dendrites and appendages of granule cells (Gr). We cannot discard sporadic synapses (?) from cholinergic axons on superficial short-axon cells (SSA). Synaptic contacts from cholinergic axons on mitral (M) and tufted (ET) cells were never found. Abbreviations: CR: type 2, calretinin-containing periglomerular cells (orange); EPL: external plexiform layer; ET: tufted cell (dark green); GCL: granule cell layer; GL: glomerular layer; Gr: granule cells (purple); M: Mitral cell (green); MCL: mitral cell layer; ON: olfactory nerve (blue). ONL: olfactory nerve layer. SSA: superficial-short-axon cell (pink); TH: type 1, tyrosine hydroxylase-containing periglomerular cells (brown).

The results shown here constitute the first report of the synaptic connectivity of the cholinergic system in the OB of primates (Figure 10). Our data demonstrate that the cholinergic axons found in the macaque OB form axo-somatic and axo-dendritic synaptic contacts on bulbar interneurons, mainly on periglomerular cells and granule cells. Although we have found VAChT-containing axons in juxtaposition with the dendrites of mitral and tufted cells, cholinergic synapses were never found on principal cells. Our data are in close agreement with those previously reported in rodents, and demonstrate that the synaptic action of the cholinergic system on the bulbar circuitry remains largely preserved between macrosmatic and microsmatic mammals.

### Identity of the Cholinergic Elements in the Macaque OB

A previous report by Porteros et al. (2007) describes the distribution of ChAT and acetylcholinesterase in the OB of the macaque under light microscopy. These authors reported that all cholinergic elements found in the bulbar layers were axons, which were identified as centrifugal axons derived from neurons located caudally to the OB. Moreover, they did not find intrinsic cholinergic neurons in the OB of this species. Our results are in close keeping with these data since a high density of cholinergic axons was found together with the lack of intrinsic cholinergic neurons.

The presence of intrinsic cholinergic neurons in the OB of mammals still remains somewhat controversial in the literature. The vast majority of investigations that have analyzed the presence of cholinergic elements in the OB of mammals have not found intrinsic cholinergic neurons in this brain area, neither in rats (Godfrey et al., 1980; Nickell and Shipley, 1988; Le Jeune and Jourdan, 1991; Butcher et al., 1992; Arvidsson et al., 1997; Ichikawa et al., 1997; Gómez et al., 2005) nor in mice (Salcedo et al., 2011), hamsters (Macrides et al., 1981), rabbits (Chao et al., 1982), hedgehogs (Crespo et al., 1999) monkeys (Porteros et al., 2007; Mundiñano et al., 2013) or humans (Mundiñano et al., 2013). However, three studies of Ojima et al. (1988), Phelps et al. (1992) and Kasa et al. (1995) respectively, have shown the presence of a few ChAT-immunopositive neurons in the OB of the rat. The relevance of these putative cholinergic neurons in the bulbar circuitry has been considered insignificant, even negligible, because of their low number (Ojima et al., 1988). Contrary to these data, a recent article by Krosnowski et al. (2012) has described the presence of a significant number of intrinsic cholinergic interneurons in the OB of C57BL/6 background transgenic mice. Krosnowski et al. (2012) reported that most of the cholinergic interneurons were periglomerular cells. For this reason, we have analyzed carefully the glomerular layer of the macaque, in order to investigate whether it contained VAChT-immunopositive periglomerular cells. As we have described in the results, we have found no somata of bulbar neurons immunostained for VAChT. However, the lack of stained somata should not be considered as a definitive argument in order to conclude that the macaque OB does not contain intrinsic cholinergic periglomerular cells. It may be the case that VAChT is not detectable by immunohistochemical techniques in the cell bodies of these neurons, but it might be detectable in their synaptic profiles. Periglomerular cells have two types of synaptic profiles. On the one hand, they have spine-like appendages in their dendrites, which are engaged in dendro-dendritic reciprocal synapses with mitral and tufted cells. These appendages are called *“gemmules”* according Rall et al. (1966) and the seminal articles by Pinching and Powell (1971a,b). The *“gemmules”* of the periglomerular cells are filled of large flattened synaptic vesicles and make symmetrical synaptic contacts on the dendrites of mitral and tufted cells. In turn, they receive asymmetrical synaptic contacts from the dendrites of mitral and tufted cells (Pinching and Powell, 1971a,b). On the other hand, periglomerular cells have axon terminals, which are also filled with large flattened vesicles and make synaptic contacts mainly on the dendrites of mitral and tufted cells (Pinching and Powell, 1971a,b). We have analyzed in detail the ultrastructure and the synaptic connectivity of the VAChT-containing puncta found in the glomerular layer of the macaque. None of them was engaged on reciprocal synaptic contacts. Moreover, none of them formed synaptic contacts on mitral and tufted cells. Finally, all the VAChT-containing puncta that we have analyzed contained round synaptic vesicles.

Concerning the presence of putative cholinergic interneurons other than periglomerular cells in the macaque OB, our results suggest that there are neither cholinergic granule cells nor cholinergic short-axon cells. Two points lead us to this statement. First, it is widely known that granule cells are anaxonic interneurons that establish dendro-dendritic reciprocal synapses with principal cells in the external plexiform layer. None of the VAChT-containing puncta found in the external plexiform layer was engaged in reciprocal synapses with principal cells. Second, a typical feature of the axon terminals of the short-axon cells is that they contain small flattened vesicles (Pinching and Powell, 1971a,b) and all VAChT-containing axons that we have found in the macaque OB contained medium-sized and large round vesicles.

The presence of putative cholinergic neurons in the OB of the macaque cannot be fully discarded. However, clear evidence supporting their existence was not found in our study. Whatever the case, the present results indicate that, if present, the cholinergic bulbar neurons should represent an insignificant percentage of the cholinergic elements that participate in the circuitry of the macaque OB.

### Identity of the Targets of the Cholinergic Axons in the Glomerular Layer

To our knowledge, the present report describes for the first time the synaptic connectivity of the cholinergic system in the circuits of the OB of primates. To establish the identity of the targets of the cholinergic axons, we have analyzed their ultrastructural features and their synaptic relationships.

The bulbar layer receiving the densest innervation by cholinergic axons is the glomerular layer. Within this layer, we have demonstrated that the cholinergic axons form perisomatic synapses on periglomerular cells. Moreover, we have demonstrated that the cholinergic fibers innervate the cell bodies of both types 1 and 2 periglomerular cells, which were identified neurochemically using the markers TH and CR as previously reported (Liberia et al., 2013). The perisomatic innervation of periglomerular cells by cholinergic axons has also been reported in the rat by Le Jeune and Jourdan (1993) and Kasa et al. (1995).

Besides axo-somatic synapses, we have found axo-dendritic synapses in the glomerular layer. Three types of dendrites participate in the circuitry of the glomerular layer: The dendrites of the principal cells, the dendrites of the periglomerular cells and the dendrites of the superficial short-axon cells. Therefore the three types were considered as potential target candidates of the cholinergic axons in this stratum. Our results showed that the dendrites receiving cholinergic synapses belong to interneurons, mainly to periglomerular cells. Three findings support this statement. First, the dendrites of the principal cells can be easily identified under electron microscopy: They have fairly regular outlines, pale cytoplasm, spherical synaptic vesicles and make asymmetrical synaptic contacts on the dendrites of periglomerular cells (Pinching and Powell, 1971a,b). None of the dendrites that were identified as belonging to principal cells were found receiving synapses from VAChT-containing boutons. Second, some dendritic shafts receiving cholinergic synapses in the periglomerular region of the glomerular layer were reconstructed back to their parent cell body using series of ultrathin sections. All the analyzed dendrites arose from the somata of periglomerular cells. Third, many of the cholinergic synapses analyzed in the glomerular layer were found on appendages similar to spines that received a second synaptic contact from a non-cholinergic axon. Interestingly, it has been described that the dendrites of the periglomerular cells have spines that can receive two synaptic contacts (Pinching and Powell, 1971a,b).

In addition to the periglomerular cells, the glomerular layer contains a second type of interneurons: The superficial short-axon cells. These interneurons constitute a sparse population of cells that extend their dendrites throughout the periglomerular region of the glomerular layer and do not innervate the neuropil of the olfactory glomeruli. They cannot be ruled out as putative targets of the cholinergic axons. On the one hand, their dendrites have some ultrastructural features that are similar to those of the dendrites of the periglomerular cells (Pinching and Powell, 1971a,b). On the other hand, some superficial short-axon cells receive synapses from cholinergic axons in the rat OB (Le Jeune and Jourdan, 1993; Kasa et al., 1995). Accordingly, our data suggested that some of the axo-dendritic cholinergic synapses found in the periglomerular region of the glomerular layer might indeed contact dendrites of superficial short-axon cells.

The potential innervation of the external tufted cells by cholinergic axons currently is the focus of an ongoing debate (Le Jeune and Jourdan, 1993; Kasa et al., 1995). Le Jeune and Jourdan (1993) reported that some external tufted cells located in the periglomerular region of a restricted set of “atypical glomeruli” were innervated by cholinergic fibers in the rat OB. However, Kasa et al. (1995) did not find synapses from cholinergic fibers on the external tufted cells of this species. Our data are in close agreement with those reported by Kasa et al. (1995) since we have not found synapses from VAChT-containing puncta on the external tufted cells of the macaque OB. Indeed, when the VAChT-containing puncta were located in close proximity to the somata of the external tufted cells, there were always found thin lamellae of glia between these elements, thus avoiding the establishment of synaptic contacts (as shown in Figure 5).

### Identity of the Targets of the Cholinergic Axons in the External Plexiform Layer and in the Inframitral Region

The inframitral region of the OB includes the internal plexiform layer and the granule cell layer. In this region, the targets of the cholinergic axons were easily identified as dendrites of granule cells on the basis of their ultrastructure and connectivity (Price and Powell, 1970a,b). The synapses were found on the spines and on the dendritic shafts of the granule cells. Our data are in close agreement with the data previously reported by Kasa et al. (1995) in the rat OB. These authors described cholinergic synapses on the spines and *“gemmules”* of the granule cells. However, they did not observe cholinergic synapses on the somata of these interneurons. We have not observed them either in the macaque.

In the external plexiform layer, the targets of the cholinergic boutons were easily identified as spines of granule cells. In this layer, we have found cholinergic axons running in close apposition to the dendrites of the mitral and tufted cells, although we have not found synaptic contacts between these elements. Identical data have been reported in the rat OB (Kasa et al., 1995). Kasa et al. hypothesized that the close apposition of the cholinergic boutons with the dendrites of the principal cells might indicate that the cholinergic system modulate the activity of the principal cells by non-synaptic release of acetylcholine.

### Functional Implications

From a comparative perspective, there are no significant differences between the synaptic connectivity of the cholinergic axons in the OB of rodents and primates. Therefore, we can assume that the cholinergic circuits play identical roles modulating the processing of the olfactory information in both groups of mammals. Electrophysiological data indicate that the cholinergic system can play multiple and opposing roles in the OB (Castillo et al., 1999; Ghatpande et al., 2006; Pressler et al., 2007; Pignatelli and Belluzzi, 2008; Tsuno et al., 2008; Ghatpande and Gelperin, 2009; D’Souza and Vijayaraghavan, 2012; Ma and Luo, 2012; D’Souza et al., 2013; Zhan et al., 2013; Rothermel et al., 2014). The multiple effects of acetylcholine on bulbar neurons can be analyzed in three different ways: The role of the neurotransmitter acting on principal cells, its effects acting on periglomerular cells, and its role acting on granule cells.

Several authors agreed that the activation of the cholinergic system excites principal cells (Castillo et al., 1999; D’Souza and Vijayaraghavan, 2012; D’Souza et al., 2013; Zhan et al., 2013; Rothermel et al., 2014). This action is mediated by nicotinic acetylcholine receptors (Castillo et al., 1999), mainly by those of the α3β4 subtype (D’Souza and Vijayaraghavan, 2012; D’Souza et al., 2013). Although nicotinic acetylcholine receptors are ionotropic, our data in monkey and those previously reported in rat (Kasa et al., 1995) do not support the existence of a synaptic action from cholinergic axons on mitral and tufted cells. However, the close apposition between the cholinergic axons and the dendrites of the principal cells suggests that the cholinergic system may modulate the excitation of principal cells by non-synaptic release of acetylcholine (Kasa et al., 1995; present data).

The action of acetylcholine on periglomerular cells is diverse as demonstrated by previous reports. On the one hand, the activation of nicotinic acetylcholine receptors excites periglomerular cells (Castillo et al., 1999). On the other hand, the activation of basal forebrain cholinergic neurons and the activation of muscarinic acetylcholine receptors induce an inhibitory response of periglomerular cells (Pignatelli and Belluzzi, 2008; Ma and Luo, 2012). Pignatelli and Belluzzi (2008) demonstrate in their article that acetylcholine inhibits dopaminergic periglomerular cells by activating metabotropic muscarinic receptors, probably those of the m2 subtype. These data are supported by anatomical studies that demonstrate the expression of m2 muscarinic receptors in the rat OB (Crespo et al., 2000). The anatomical data that we have shown here, support the existence of a direct synaptic action of cholinergic axons on periglomerular cells, including those that express TH and, therefore, are dopaminergic. Further studies should clarify whether the nicotinic and/or the muscarinic acetylcholine receptors mediate the synaptic transmission on these interneurons.

Finally, some published data indicate that acetylcholine can modulate the excitability of granule cells and the neurotransmitter release at the dendro-dendritic synaptic contacts between these interneurons and principal cells (Castillo et al., 1999; Ghatpande et al., 2006; Pressler et al., 2007; Tsuno et al., 2008; Ghatpande and Gelperin, 2009). Our data demonstrate that these effects may be mediated by synaptic mechanisms in the external plexiform layer and in the inframitral region of the monkey OB.

Data reported in this article indicate that the pattern of synaptic connectivity of the cholinergic axons is conserved between macrosmatic and microsmatic animals. This fact suggests that the cholinergic circuits could play a key role modulating the processing of the olfactory information in the OB of mammals. Since cholinergic circuits are affected in some neurodegenerative disorders, such as Alzheimer disease, our data could be helpful to investigate in the future whether the hyposmia associated to this disease is linked to the loss of cholinergic synaptic transmission in the OB.

## Conflict of Interest Statement

The authors declare that the research was conducted in the absence of any commercial or financial relationships that could be construed as a potential conflict of interest.

## References

[B1] Arvidsson U. Riedl M. Elde R. Meister B. (1997). Vesicular acetylcholine transporter (VAChT) protein: a novel and unique marker for cholinergic neurons in the central and peripheral nervous systems. J. Comp. Neurol. 378, 454–467. 10.1002/(SICI)1096-9861(19970224)378:4<454::AID-CNE2>3.0.CO;2-1 9034903

[B2] Broadwell R. D. Jacobowitz D. M. (1976). Olfactory relationships of the telencephalon and diencephalon in the rabbit. III. The ipsilateral centrifugal fibers to the olfactory bulbar and retrobulbar formations. J. Comp. Neurol. 170, 321–345. 10.1002/cne.901700305 62770

[B3] Butcher L. L. Oh J. D. Woolf N. J. Edwards R. H. Roghani A. (1992). Organization of central cholinergic neurons revealed by combined *in situ* hybridization histochemistry and choline-O-acetyltransferase immunocytochemistry. Neurochem. Int. 21, 429–445. 10.1016/0197-0186(92)90195-w 1303168

[B4] Carson K. A. (1984). Localization of acetylcholinesterase-positive neurons projecting to the mouse main olfactory bulb. Brain Res. Bull. 12, 635–639. 10.1016/0361-9230(84)90144-8 6206927

[B5] Carson K. A. Burd G. D. (1980). Localization of acetylcholinesterase in the main and accessory olfactory bulbs of the mouse by light and electron microscopic histochemistry. J. Comp. Neurol. 191, 353–371. 10.1002/cne.901910304 7410598

[B6] Castillo P. E. Carleton A. Vincent J. D. Lledo P. M. (1999). Multiple and opposing roles of cholinergic transmission in the main olfactory bulb. J. Neurosci. 19, 9180–9191. 10531421 10.1523/JNEUROSCI.19-21-09180.1999 PMC6782910

[B7] Chao L. P. Kan K. S. Hung F. M. (1982). Immunohistochemical localization of choline acetyltransferase in rabbit forebrain. Brain Res. 235, 65–82. 10.1016/0006-8993(82)90196-2 6765220

[B8] Crespo C. Blasco-Ibáñez J. M. Briñón J. G. Alonso J. R. Domínguez M. I. Martínez-Guijarro F. J. (2000). Subcellular localization of m2 muscarinic receptors in GABAergic interneurons of the olfactory bulb. Eur. J. Neurosci. 12, 3963–3974. 10.1046/j.1460-9568.2000.00289.x 11069592

[B9] Crespo C. Briñón J. G. Porteros A. Arévalo R. Rico B. Aijón J.. (1999). Distribution of acetylcholinesterase and choline acetyltransferase in the main and accessory olfactory bulbs of the hedgehog (*Erinaceus europaeus*). J. Comp. Neurol. 403, 53–67. 10.1002/(sici)1096-9861(19990105)403:1<53::aid-cne5>3.0.co;2-o 10075443

[B10] D’Souza R. D. Parsa P. V. Vijayaraghavan S. (2013). Nicotinic receptors modulate olfactory bulb external tufted cells via an excitation-dependent inhibitory mechanism. J. Neurophysiol. 110, 1544–1553. 10.1152/jn.00865.2012 23843430 PMC4042413

[B11] D’Souza R. D. Vijayaraghavan S. (2012). Nicotinic receptor-mediated filtering of mitral cell responses to olfactory nerve inputs involves the α3β4 subtype. J. Neurosci. 32, 3261–3266. 10.1523/jneurosci.5024-11.2012 22378897 PMC3306821

[B12] Ghatpande A. S. Gelperin A. (2009). Presynaptic muscarinic receptors enhance glutamate release at the mitral/tufted to granule cell dendrodendritic synapse in the rat main olfactory bulb. J. Neurophysiol. 101, 2052–2061. 10.1152/jn.90734.2008 19225175

[B13] Ghatpande A. S. Sivaraaman K. Vijayaraghavan S. (2006). Store calcium mediates cholinergic effects on mIPSCs in the rat main olfactory bulb. J. Neurophysiol. 95, 1345–1355. 10.1152/jn.00757.2005 16319214

[B14] Godfrey D. A. Ross C. D. Herrmann A. D. Matschinsky F. M. (1980). Distribution and derivation of cholinergic elements in the rat olfactory bulb. Neuroscience 5, 273–292. 10.1016/0306-4522(80)90103-7 7374942

[B15] Gómez C. Briñón J. G. Barbado M. V. Weruaga E. Valero J. Alonso J. R. (2005). Heterogeneous targeting of centrifugal inputs to the glomerular layer of the main olfactory bulb. J. Chem. Neuroanat. 29, 238–254. 10.1016/j.jchemneu.2005.01.005 15927786

[B16] Ichikawa T. Ajiki K. Matsuura J. Misawa H. (1997). Localization of two cholinergic markers, choline acetyltransferase and vesicular acetylcholine transporter in the central nervous system of the rat: *in situ* hybridization histochemistry and immunohistochemistry. J. Chem. Neuroanat. 13, 23–39. 10.1016/s0891-0618(97)00021-5 9271193

[B17] Kasa P. Hlavati I. Dobo E. Wolff A. Joo F. Wolff J. R. (1995). Synaptic and non-synaptic cholinergic innervation of the various types of neurons in the main olfactory bulb of adult rat: immunocytochemistry of choline acetyltransferase. Neuroscience 67, 667–677. 10.1016/0306-4522(95)00031-d 7675193

[B18] Kimura H. McGeer P. L. Peng J. H. McGeer E. G. (1981). The central cholinergic system studied by choline acetyltransferase immunohistochemistry in the cat. J. Comp. Neurol. 200, 151–201. 10.1002/cne.902000202 7287919

[B19] Kosaka K. Toida K. Margolis F. L. Kosaka T. (1997). Chemically defined neuron groups and their subpopulations in the glomerular layer of the rat main olfactorybulb–II. Prominent differences in the intraglomerular dendritic arborization and their relationship to olfactory nerve terminals. Neuroscience 76, 775–786. 10.1016/s0306-4522(96)00308-9 9135050

[B20] Kovacs I. Torok I. Zombori J. Kasa P. (1998). Cholinergic structures and neuropathologic alterations in the olfactory bulb of Alzheimer’s disease brain samples. Brain Res. 789, 167–170. 10.1016/s0006-8993(98)00097-3 9602111

[B21] Krosnowski K. Ashby S. Sathyanesan A. Luo W. Ogura T. Lin W. (2012). Diverse populations of intrinsic cholinergic interneurons in the mouse olfactory bulb. Neuroscience 213, 161–178. 10.1016/j.neuroscience.2012.04.024 22525133 PMC3367073

[B22] Le Jeune H. Jourdan F. (1991). Postnatal development of cholinergic markers in the rat olfactory bulb: a histochemical and immunocytochemical study. J. Comp. Neurol. 314, 383–395. 10.1002/cne.903140212 1787181

[B23] Le Jeune H. Jourdan F. (1993). Cholinergic innervation of olfactory glomeruli in the rat: an ultrastructural immunocytochemical study. J. Comp. Neurol. 336, 279–292. 10.1002/cne.903360209 8245219

[B24] Liberia T. Blasco-Ibáñez J. M. Nácher J. Varea E. Lanciego J. L. Crespo C. (2013). Two types of periglomerular cells in the olfactory bulb of the macaque monkey (*Macaca fascicularis*). Brain Struct. Funct. 218, 873–887. 10.1007/s00429-012-0435-0 22684581

[B25] Ma M. Luo M. (2012). Optogenetic activation of basal forebrain cholinergic neurons modulates neuronal excitability and sensory responses in the main olfactory bulb. J. Neurosci. 32, 10105–10116. 10.1523/jneurosci.0058-12.2012 22836246 PMC6703727

[B26] Macrides F. Davis B. J. Youngs W. M. Nadi N. S. Margolis F. L. (1981). Cholinergic and catecholaminergic afferents to the olfactory bulb in the hamster: a neuroanatomical, biochemical and histochemical investigation. J. Comp. Neurol. 203, 495–514. 10.1002/cne.902030311 6274923

[B27] Mesulam M. M. Mufson E. J. Levey A. I. Wainer B. H. (1983). Cholinergic innervation of cortex by the basal forebrain: cytochemistry and cortical connections of the septal area, diagonal band nuclei, nucleus basalis (substantia innominata) and hypothalamus in the rhesus monkey. J. Comp. Neurol. 214, 170–197. 10.1002/cne.902140206 6841683

[B28] Mundiñano I. C. Hernandez M. Dicaudo C. Ordoñez C. Marcilla I. Tuñon M. T.. (2013). Reduced cholinergic olfactory centrifugal inputs in patients with neurodegenerative disorders and MPTP-treated monkeys. Acta Neuropathol. 126, 411–425. 10.1007/s00401-013-1144-3 23784261

[B29] Nickell W. T. Shipley M. T. (1988). Two anatomically specific classes of candidate cholinoceptive neurons in the rat olfactory bulb. J. Neurosci. 8, 4482–4491. 3199188 10.1523/JNEUROSCI.08-12-04482.1988 PMC6569571

[B30] Ojima H. Yamasaki T. Kojima H. Akashi A. (1988). Cholinergic innervation of the main and the accessory olfactory bulbs of the rat as revealed by a monoclonal antibody against choline acetyltransferase. Anat. Embryol. (Berl) 178, 481–488. 10.1007/bf00305035 3223607

[B31] Phelps P. E. Houser C. R. Vaughn J. E. (1992). Small cholinergic neurons within fields of cholinergic axons characterize olfactory-related regions of rat telencephalon. Neuroscience 48, 121–136. 10.1016/0306-4522(92)90343-z 1584418

[B32] Pignatelli A. Belluzzi O. (2008). Cholinergic modulation of dopaminergic neurons in the mouse olfactory bulb. Chem. Senses 33, 331–338. 10.1093/chemse/bjm091 18209017

[B33] Pinching A. J. Powell T. P. (1971a). The neuron types of the glomerular layer of the olfactory bulb. J. Cell Sci. 9, 305–345. 4108056 10.1242/jcs.9.2.305

[B34] Pinching A. J. Powell T. P. (1971b). The neuropil of the glomeruli of the olfactory bulb. J. Cell Sci. 9, 347–377. 4108057 10.1242/jcs.9.2.347

[B35] Porteros A. Arévalo R. Weruaga E. Crespo C. Briñón J. G. Alonso J. R.. (1997). Calretinin immunoreactivity in the developing olfactory system of the rainbow trout. Brain Res. Dev. Brain Res. 100, 101–109. 10.1016/s0165-3806(97)00037-0 9174251

[B36] Porteros A. Gómez C. Valero J. Calvo-Baltanás F. Alonso J. R. (2007). Chemical organization of the macaque monkey olfactory bulb: III. Distribution of cholinergic markers. J. Comp. Neurol. 501, 854–865. 10.1002/cne.21280 17311313

[B37] Pressler R. T. Inoue T. Strowbridge B. W. (2007). Muscarinic receptor activation modulates granule cell excitability and potentiates inhibition onto mitral cells in the rat olfactory bulb. J. Neurosci. 27, 10969–10981. 10.1523/jneurosci.2961-07.2007 17928438 PMC6672850

[B38] Price J. L. Powell T. P. (1970a). The morphology of the granule cells of the olfactory bulb. J. Cell Sci. 7, 91–123. 5476864 10.1242/jcs.7.1.91

[B39] Price J. L. Powell T. P. (1970b). The synaptology of the granule cells of the olfactory bulb. J. Cell Sci. 7, 125–155. 5476853 10.1242/jcs.7.1.125

[B40] Rall W. Shepherd G. M. Reese T. S. Brightman M. W. (1966). Dendrodendritic synaptic pathway for inhibition in the olfactory bulb. Exp. Neurol. 14, 44–56. 10.1016/0014-4886(66)90023-9 5900523

[B41] Rothermel M. Carey R. M. Puche A. Shipley M. T. Wachowiak M. (2014). Cholinergic inputs from Basal forebrain add an excitatory bias to odor coding in the olfactory bulb. J. Neurosci. 34, 4654–4664. 10.1523/jneurosci.5026-13.2014 24672011 PMC3965788

[B42] Rye D. B. Wainer B. H. Mesulam M. M. Mufson E. J. Saper C. B. (1984). Cortical projections arising from the basal forebrain: a study of cholinergic and noncholinergic components employing combined retrograde tracing and immunohistochemical localization of choline acetyltransferase. Neuroscience 13, 627–643. 10.1016/0306-4522(84)90083-6 6527769

[B43] Salcedo E. Tran T. Ly X. Lopez R. Barbica C. Restrepo D.. (2011). Activity-dependent changes in cholinergic innervation of the mouse olfactory bulb. PLoS One 6:e25441. 10.1371/journal.pone.0025441 22053179 PMC3203864

[B44] Tsuno Y. Kashiwadani H. Mori K. (2008). Behavioral state regulation of dendrodendritic synaptic inhibition in the olfactory bulb. J. Neurosci. 28, 9227–9238. 10.1523/jneurosci.1576-08.2008 18784303 PMC6670912

[B45] Weruaga E. Briñón J. G. Porteros A. Arévalo R. Aijón J. Alonso J. R. (2001). A sexually dimorphic group of atypical glomeruli in the mouse olfactory bulb. Chem. Senses 26, 7–15. 10.1093/chemse/26.1.7 11124210

[B46] Záborszky L. Carlsen J. Brashear H. R. Heimer L. (1986). Cholinergic and GABAergic afferents to the olfactory bulb in the rat with special emphasis on the projection neurons in the nucleus of the horizontal limb of the diagonal band. J. Comp. Neurol. 243, 488–509. 10.1002/cne.902430405 3512629

[B47] Zhan X. Yin P. Heinbockel T. (2013). The basal forebrain modulates spontaneous activity of principal cells in the main olfactory bulb of anesthetized mice. Front. Neural Circuits 7:148. 10.3389/fncir.2013.00148 24065892 PMC3778317

[B48] Zheng L. M. Ravel N. Jourdan F. (1987). Topography of centrifugal acetylcholinesterase-positive fibres in the olfactory bulb of the rat: evidence for original projections in atypical glomeruli. Neuroscience 23, 1083–1093. 10.1016/0306-4522(87)90183-7 3437990

